# Bipolar hemiarthroplasty may reduce cerebrovascular accidents and improve early weight-bearing in the elderly after femoral neck fracture

**DOI:** 10.1097/MD.0000000000028635

**Published:** 2022-01-28

**Authors:** Jeremy Dubin, Ran Atzmon, Viktor Feldman, Uri Farkash, Meir Nyska, Ehud Rath, Esequiel Palmanovich

**Affiliations:** a Tel Aviv Medical Center, Department of Orthopaedic Surgery, Affiliated with the Sackler Faculty of Medicine and Tel Aviv University, Tel Aviv, Israel; b Assuta Medical Center, Department of Orthopaedic Surgery, Affiliated with the Faculty of Health and Science and Ben Gurion University, Ashdod, Israel; c Meir Hospital, Orthopedic Department, Sapir Medical Center, Kfar Saba, Israel. Affiliated with the Sackler Faculty of Medicine, Tel Aviv University, Tel Aviv, Israel.

**Keywords:** bipolar hemiarthroplasty, cannulated screw, cerebrovascular accident, elderly population, hip fracture, weight bearing

## Abstract

Cerebrovascular accidents (CVA) in the elderly population after femoral neck fracture remain great concern for physicians. Specifically, surgical fixation techniques, such as bipolar hemiarthroplasty (HA) and internal fixation play a significant role in influencing the occurrence of postoperative CVA in the elderly population.

In order to identify 2 cohorts, we used a rigid selection process based on our institution's database. The cohorts were comprised of a HA cohort and a cannulated screw cohort, of which underwent femoral neck surgery, performed by 3 fellowship trained surgeons from 2003 to 2014. Risk factors were documented and measured, including Coumadin use and hypertension, and postoperative complications such as CVA and death rate were also recorded. A *P*-value of <.05 was determined to be statistically significant.

A power analysis was performed and achieved a power of 0.95. We found a non-significant reduction in CVA for bipolar HA (3.6% CVA vs 0.0% in the non-CVA group, *P* = .48) and a non-significant increase in CVA for cannulated screw use (7.6% CVA vs 14.4% in the non-CVA group, *P* = .11). In addition, we found a significant difference in terms of weight-bearing status at 6-weeks postoperatively (0.95 vs 2.0, *P* < .0001), favoring the bipolar HA group.

Among the advantages of bipolar HA surgery, surgeons should consider its value in reducing the occurrence of postoperative CVA. Furthermore, patients who underwent bipolar HA had improved weight-bearing status postoperatively compared with cannulated screw fixation.

## 1 Introduction

In 2010, 158 million people 50 years or older had a fracture probability at or above high-risk threshold. By 2040, the number of individuals is expected to double to approximately 319 million.^[1]^ Moreover, according to the literature, 1 in 3 adults aged 50 and over, dies within 12 months of suffering a hip fracture.^[2]^ The majority of the patients do not return to their pre-fracture leisure and daily activities, and find it difficult to restore their quality of life. Furthermore, it was shown that the fracture may even jeopardize the patients’ mental health as well.^[3]^ Potential complications of proximal hip fractures include; blood clots formation, decubitus ulcers, urinary tract infections, pneumonia, and death as a result of diminished daily activities and bedridden patients.^[4]^ Given the disruption of the blood supply at the femoral neck, the healing ability of the fracture may be compromised, and may lead to fracture non-union as a result of avascular necrosis or osteonecrosis. Another important consequence is a postoperative cerebrovascular accidents (CVA). Patients who suffered a CVA after a hip fracture have an increased risk of mortality relative to patients without CVA.^[5]^

Several treatment options exist for hip fractures, which are dependent on the location of the fracture, bone stability, patients’ daily activities and requirements, and age of the patient. The treatment for femoral neck fractures has been controversial throughout the literature on the basis that internal fixation can lead to high rates of fixture failures and nonunion, leading to a shift in treatment using arthroplasty options.^[6,7]^ One guideline recommended the division of femoral neck fracture as follows; screw fixation for nondisplaced fracture, HA for displaced fracture with low activity patients, and total hip arthroplasty for high activity patients.^[8]^ On the other hand, treatment for intertrochanteric fractures remains clearer and has been divided into sliding hip screw and side plate for stable fractures, and intramedullary hip screw for unstable fractures.^[8]^

Common treatments for fractures of the femoral neck are HA or internal fixation (IF) particularly with cannulated screw fixation.^[9]^ The benefits of a bipolar HA include; improved stability in elderly patients, immediate weight bearing, and lower reoperation rate among other advantages.^[10,11]^ However, complications include; open surgical procedure which might lead to surgical site infection and an increase blood loss, stem loosening, periprosthetic fracture, and prosthetic head dislocation.^[12]^ On the other hand, IF benefits from less operative time, lower risk of infection, closed surgical procedure which does not jeopardies the stability of the hip, and potentially lower early mortality rate.^[13]^ However, IF is associated with higher incidence of reoperation and non-union.^[14]^

The relationship between the type of surgical fixation method after proximal femoral fractures and postoperative CVA in the elderly population is limited in the literature. Understanding the relationship between a relevant risk factors in a high-risk population, provides a desirable impetus for pursuit of this study. We hypothesized that bipolar HA would have lower incidence of CVA in the elderly population in relation to IF, a higher level of weight bearing status, but a higher mortality rate postoperatively at 3-year follow-up compared with cannulated screw fixation at a minimum follow-up of 24 months.

## 2 Methods

**Figure 1:**
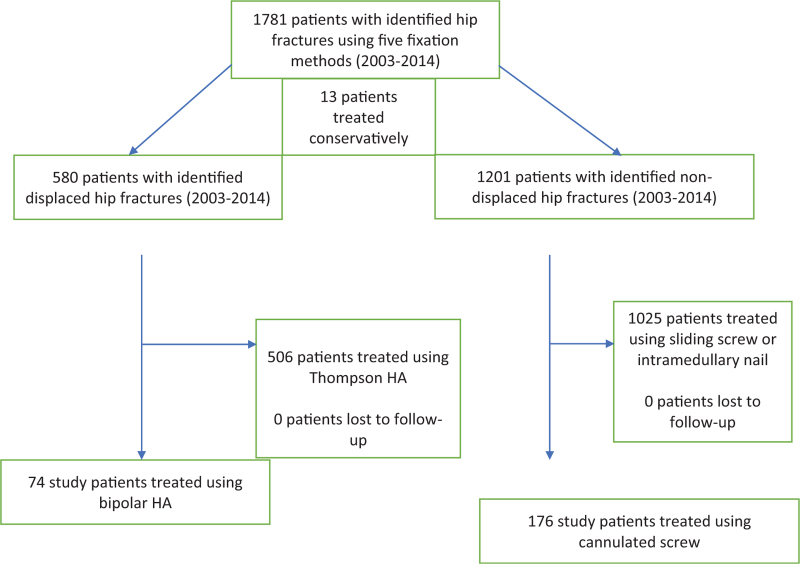
Flow chart of patient selection, including patients identified with hip fractures using 5 fixation methods and patients treated conservatively.

We performed a retrospective review of prospectively collected data in our institution's database and identified patients who presented to a level 3 trauma center with hip fractures between 2003 and 2014 with a minimum follow-up of 24 months (Fig. 1). This was 1 of 4 different studies using the database with a focus on HA and IF in the current analysis. Additional fixation methods were included to comparing the influence of fixation method on postoperative incidence of CVA (Table 1). Review was done via a questionnaire and radiographic analysis of offset by independent observers. The fractures were analyzed and classified using the Pauwel Classification and Garden Classification, which categorize femoral fractures in term of vertical orientation of fracture line and categorization based on anterior-posterior (AP) radiographs, respectively. A rigid selection process was used to determine usage of either the bipolar HA procedure or the cannulated screw procedure. Our inclusion criteria included: a minimum 2-years follow-up period, and patients who were diagnosed with femoral neck fractures according to the International Classification of Diseases, 9^th^ Revision, Clinical Modifications codes 820-821. Our exclusion criteria included: <2-year follow-up, pathological fractures, conservatively treated hip fractures, and lack of medical records that prevented classification.

**Table 1:** Postoperative CVA based on fixation type, including bipolar HA, cannulated screw, Thompson HA, sliding hip screw, and intramedullary nail.

	No CVA	CVA	*P*-value
Bipolar HA	71 (3.6%)	0 (0%)	.048
Cannulated screw	150 (7.6%)	15 (8.0%)	.011
Thompson HA	482 (24.3%)	24 (23.1%)	.775
Sliding hip screw	118 (6.1%)	5 (4.8%)	.629
Intramedullary nail	121 (6.1%)	6 (5.8%)	.819

CVA = cerebrovascular accidents, HA = hemiarthroplasty.

### 2.1 Bipolar HA procedure

A posterior approach was utilized with an 18 to 20 cm incision at the greater trochanter. Then, the fascia lata is cut distally, proximally, and then retracted to create a D-shape towards to the knee. The abductors are detached from the greater trochanter using a diathermy in a s-shape to reveal the capsule. An oscillating saw is used at a 45° angle to make the neck cut, the femoral head is removed and measured using the guide. The head is trialed, a cement restrictor is inserted, and the stem insertion and head attachment are placed to have 12 to 15degrees of anteversion. AP and lateral images are taken to confirm proper placement of the implant.

### 2.2 Cannulated screw procedure

A direct lateral approach is taken with a 4 to 5 mm incision starting distally at the tip of the greater trochanter was used. Three guidewires are inserted to form an inverted triangle with 2 screws proximally and 1 distally. The first guidewire is inserted above the calcar and reaches the subchondral bone in the femoral head. The second guidewire is placed superoposterior to the first guidewire while the third guidewire is placed superoanterior to the first guidewire. The appropriate measurement of the screw length is made. A cannulated drill is placed 7 to 10-mm short of the measured distanced and used over the guidewires. Three screws and washers are inserted and tightened. AP and lateral images are taken to confirm proper placement of the screws.

### 2.3 Postoperative protocol

The postoperative protocols were uniform in both cohorts. At admission, and for at least 8 to 10 days postoperatively, all patients received a subcutaneous injection of 40 mg of Enoxaparin for venous thromboembolic disease prophylaxis. However, patients who had a pre-existing risk for thromboembolic event, such as previous pulmonary embolism, cerebrovascular accident, or adverse event received 60 to 90 mg twice a day. Patients in both groups were given a walker or 2 crutches postoperatively, and precautions to avoid dislocation of the prosthesis, including avoiding movement beyond 90° from a seated position and not crossing the legs while sleeping. At 6 weeks postoperatively, patients were mobilized full weight without any other restrictions. Patients were recommended to undergo rehabilitation under the physiotherapists’ protocol. The subsequent postoperative visits were performed at 3, 6, and 12 months, which included oblique and AP standing radiographs.

### 2.4 Data collection

Data were collected by the physician at the preoperative visit and subsequent postoperative visits. Data collected included sex, age, fracture type, fixation type, time interval from admission to operation, time interval from operation to discharge, postoperative weight bearing status, and rehabilitation protocol. In addition, past medical history that was recorded included diabetes mellitus (DM), hypertension (HTN), hypercholesterolemia (HCL), ischemic heart disease (IHD), atrial fibrillation (AF), congestive heart failure (CHF), chronic renal failure (CRF), pulmonary embolism (PE), and deep vein thrombosis (DVT). The study was approved by the Weizmann Institute's Institutional Review Board.

### 2.5 Statistical analysis

All statistical analyses were performed using R, from the R Core Team (2015). R: A language and environment for statistical computing. R Foundation for Statistical Computing, Vienna, Austria. URL https://www.R-project.org/. The chi-squared test was used for calculating the incidence of CVA based on the sample size (Table 1). Normally distributed continuous data was compared using the Student *t* test data. A power analysis was performed to determine the appropriate number of patients in each cohort to achieve a desired power of 0.95. A *P*-value of <.05 was determined to be statistically significant.

## 3 Results

A total of 2400 patients underwent hip fracture surgery in the period of 11 years between 2003 and 2014. There were 1781 patients identified in this study using 5 principal fixation methods (Fig. 1). The bipolar HA cohort was the only surgical fixation types that showed significant difference between the occurrence of postoperative CVA (0.0% vs 3.3% in the other fixation methods) (*P* = .048). On the other hand, the cannulated screw cohort was the only surgical fixation type that showed a significant increase the occurrence of postoperative CVA versus no CVA postoperatively cohort (14.4% no CVA vs 7.6% CVA, *P* = .011) (Table 1). A power analysis revealed the sample size in each cohort to obtain a power of 0.95 must be n = 60. In our analysis, there were 74 patients in the bipolar HA cohort and 176 patients in the cannulated screw cohort (Table 2).

**Table 2:** Patient demographics, including number in each cohort, average, and males to females breakdown.

	Bipolar HA	Cannulated screw	*P*-value
Number in each cohort	74	176	N/A
Average age	80.29	80.66	.80
Males: females	27:47	54:122	.39

HA = hemiarthroplasty.

We found no significant difference between the 2 groups in terms of risk factors, including Coumadin use (*P* = .61), pulmonary embolism (*P* = N/A), deep vein thrombosis (*P* = N/A), hypertension (*P* = .17), atrial fibrillation (*P* = .76), diabetes mellitus (*P* = .30), and hypocholesteremia (*P* = .86) (Table 3). However, we did find a significant difference between the cohorts in terms of weight-bearing status at 6-weeks postoperatively (0.95 vs 2.0, *P* < .0001), favoring the bipolar HA group (Table 4).

**Table 3:** Comparison of risk factors in each cohort, including coumadin, pulmonary embolism, deep vein thrombosis, hypertension, atrial fibrillation, diabetes mellitus, hypocholesteremia.

	Bipolar HA No = 0 Yes = 1	Cannulated screw No = 0 Yes = 1	*P*-value
Coumadin	0.07	0.05	.61
Pulmonary embolism	0	0	NA
Deep vein thrombosis	0	0	NA
Hypertension	0.59	0.50	.17
Atrial fibrillation	0.12	0.11	.76
Diabetes mellitus	0.27	0.21	.30
Hypocholesteremia	0.24	0.23	.86

**Table 4:** Comparison of postoperative outcomes in each cohort, including rehabilitation, weight-bearing at 6 weeks’ post-op, and percent of deaths.

	Bipolar HA	Cannulated screw	*P*-value
Rehabilitation (No = 0, Yes = 1)	0.28	0.21	.21
Weight-bearing at 6 weeks’ post-op (1 = full, 2 = partial, 3 = none)	0.95	2.0	<.0001
Percent of deaths (3 years follow-up)	8%	11%	.44

HA = hemiarthroplasty.

## 4 Discussion

The main objective in the elderly remains to optimize medical comorbidities and allow early mobilization.^[7]^ This objective seems to be at odds in regards to the treatment of femoral neck fractures in the elderly.^[6]^ We examined the differences between the 2 main techniques used in femoral neck fractures’ fixation, arthroplasty, and internal fixation, in regards to postoperative outcomes at a minimum follow-up of 24 months.

We found support in our hypothesis by finding that the bipolar HA cohort had significantly lower presence of CVA (0.0%) postoperatively than other fixation type. Moreover, we showed that the bipolar HA cohort had higher weight-bearing status than the cannulated screw cohort (0.95 vs 2.0, *P* < .0001). However, we did not find a significance difference in terms of survival rate between the bipolar HA cohort and cannulated screw cohort, respectively (8% vs 11%, *P* = .44). Our study both acts to support the current literature with regards to mortality rate and weight-bearing preference for bipolar HA group, and expand the scope by finding that bipolar HA may lower the presence of CVA postoperatively.

The relationship between femoral neck fractures and postoperative CVA has been established in the literature but without distinction between type of surgical fixation and limited follow-up time periods. The finding that bipolar HA in comparison to 4 other fixation methods, significantly reduced CVA postoperatively is novel in the literature.^[15]^ Popa et al^[16]^ discovered a 3.9% probability of ischemic stroke over the first postoperative year in a cohort of 1606 patients. Lawrence et al^[17]^ found a 1.0% probability of stroke at 1 year postoperatively in a cohort of 8930 patients, respectively. One study found in 356 consecutive patients aged >70 undergoing bipolar HA had delayed recovery from surgical stress led to postoperative delirium.^[18]^ We found that improved bipolar HA had significantly improved weight-bearing status, which may explain this relationship to some extent. In addition, this analysis found that cannulated screws (IF) may be a risk factor for postoperative CVA (*P* = .011) due to its association with poor weight-bearing outcomes in the literature.^[18]^

The literature offers evidence that weight bearing protocols may favor the bipolar HA surgery over the cannulated screw fixation as we found in our study. Yang et al^[19]^ found more persistent pain, malformation, stiffness, and function limitation in the internal fixation group resulting from bed rest compared with the hemiarthroplasty group in their randomized controlled trials. In addition, they found early weight bearing protocols were favorable in the hemiarthroplasty group and minimized complications of prolonged inactivity after surgery. Gjertsen et al^[20]^ also found favorable outcomes for the bipolar hemiarthroplasty group in terms of satisfaction with operation result (38.9 vs 25.7), lower quality of life (0.51 vs 0.60), and pain (29.9 vs 19.2) compared with the internal screw fixation group in a study of 4335 patients over 70 years old at a minimum follow-up of 12 months. This mitigates the concern that bipolar HA has been shown to have a high risk of pain and operative trauma in treatment of femoral neck.^[21]^ On the other hand, Parker et al^[22]^ noted no difference in pain or mobility between the 2 groups but found that blood loss (28 mL vs 177 mL, *P* < .0001) and length of anesthesia (36 minutes vs 57 minutes, *P* < .0001) were more favorable in the IF group than the hemiarthroplasty group. They recommend the use of IF in patients who are very handicapped, which is consistent with our finding that patients in the cannulated screw group benefit from shorter hospitalization stays than the bipolar HA cohort (Table 3). In addition, this finding could be used to adapt the selection process by directing patients of this physical characteristic to the IF approach.

Our finding, which shows the mortality rate between the 2 groups (8% vs 11%, *P* = .44) is not significant and is consistent with the literature. Yang et al^[19]^ and Parker et al^[22]^ found no difference in mortality rate between the bipolar HA and IF cohorts in their meta-analysis and randomized studies (15.1% vs 12.8%, *P* = .19 and 63/229 vs 61/226, *P* = .91), respectively. Yang et al^[19]^ found a significant difference in reoperation rates at both 1 year and greater than 2 years, favoring the hemiarthroplasty group (94/1447 vs 404/1167, *P* < .001 at 1 year) and (46/276 vs 144/284, *P* < .005 at greater than 2 years), respectively. This is consistent the use of cannulated screws in highly frail elders that are part of a high-risk cohort from the start. One recent analysis showed that patients treated with HA gained 0.15 to 0.20 more quality-adjusted life years (QALYs) than patients treated with IF that was not associated with greater hospital costs.^[23]^ Another analysis showed that HA was associated with 2.96 greater QALYs than IF, which was associated with lower cost ($23,467 vs $25,356) based on the cost per QALY of $7925 for HA compared with $9303 for screw fixation.^[24]^ Cost analyses are relevant to weigh with the decreased CVA and improvement in weight-bearing status following femoral neck fracture that was established in this study.

### 4.1 Limitation

We acknowledge limitations in the study. The study would benefit from a prospective approach, in which the cohorts are of large patient numbers. However, we mitigate the concerns by performing a power analysis, which reveals a high-power level of 0.95 in our analysis and the use of a comparison group as well. In addition, a longer follow-up (>24 months) would help improve the study but the outcomes in the literature are consistent with the results we found at a minimum of 24 months and allowed us to include a large number of patients.^[12]^ We did not examine fracture type in this study, which could impact postoperative CVA but our selection process and consistency within the literature helps to validate the methodology of our current system.^[5]^ In addition, we found no significant difference in terms of risk factors between the cohorts (Table 2), which addresses additional concerns.

## 5 Conclusion

Bipolar HA use led to a significant reduction in postoperative CVA incidence. In addition, in comparing bipolar HA and cannulated screw fixation methods, patients had an improved weight-bearing status after bipolar HA fixation. However, in certain patients, such as the very fragile, cannulated screw fixation can reduce the overall hospitalization of the patients at the expense of increased CVA incidence and should be taken into consideration in future preoperative protocols.

## Author contributions

**Conceptualization:** Jeremy Dubin, Viktor Feldman, Uri Farkash, Meir Nyska, Ehud Rath, Esequiel Palmanovich.

**Data curation:** Jeremy Dubin, Ran Atzmon, Viktor Feldman, Uri Farkash, Meir Nyska, Ehud Rath, Esequiel Palmanovich.

**Formal analysis:** Jeremy Dubin, Ran Atzmon, Esequiel Palmanovich.
